# Influence of Otolaryngological Subspecialties on Perception of Transoral Robotic Surgery: An International YO-IFOS Survey

**DOI:** 10.3390/jpm13121717

**Published:** 2023-12-15

**Authors:** Antonino Maniaci, Carlos Chiesa Estomba, Nicolas Fakhry, Luigi Angelo Vaira, Marc Remacle, Giovanni Cammaroto, Maria Rosaria Barillari, Giannicola Iannella, Miguel Mayo-Yanez, Alberto Maria Saibene, Robin Baudouin, Juan Maza-Solano, Abie H. Mendelsohn, Floyd Christopher Holsinger, Fabio P. Ceccon, Leonardo Haddad, Stephane Hans, Ignazio La Mantia, Salvatore Cocuzza, Federica Gulinello, Tareck Ayad, Jerome R. Lechien

**Affiliations:** 1 Robotics Study Group of Young Otolaryngologists, International Federation of Oto-Rhino-Laryngological Societies, 75000 Paris, France; chiesaestomba86@gmail.com (C.C.E.); nicolas.fakhry@ap-hm.fr (N.F.); luigi.vaira@gmail.com (L.A.V.); giovanni.cammaroto@hotmail.com (G.C.); mariarosaria.barillari@unicampania.it (M.R.B.); giannicola.iannella@uniroma1.it (G.I.); miguel.mayo.yanez@sergas.es (M.M.-Y.); alberto.saibene@gmail.com (A.M.S.); robin.baudouin@aol.fr (R.B.); juan.maza.solano@gmail.com (J.M.-S.); tareckayad@yahoo.ca (T.A.); jerome.lechien@umons.ac.be (J.R.L.); 2 Faculty of Medicine and Surgery, University of Enna “Kore”, 94100 Enna, Italy; 3 Department of Oto-Rhino-Laryngology Head and Neck Surgery, La Conception University Hospital, AP-HM, Aix Marseille Univ, 13005 Marseille, France; 4 Department of Otorhinolaryngology-Head and Neck Surgery, Hospital Universitario Donostia, 20003 San Sebastian, Spain; 5 Maxillofacial Surgery Unit, University Hospital of Sassari, 07025 Sassari, Italy; 6 Department of Otorhinolaryngology-Head and Neck Surgery, CHL-Eich, Rue d’Eich 78, 1111 Luxembourg, Luxembourg; marc.remacle01@gmail.com; 7 Head-Neck, and Oral Surgery Unit, Department of Head-Neck Surgery, Otolaryngology, Morgagni Pierantoni Hospital, 47121 Forlì, Italy; 8 Department of Mental and Physical Health and Preventive Medicine, “L. Vanvitelli” University, 80121 Naples, Italy; 9 Organi di Senso Department, Sapienza University of Rome, Viale del Policlinico 151, 00161 Rome, Italy; 10 Otorhinolaryngology-Head and Neck Surgery Department, Complexo Hospitalario Universitario A Coruña (CHUAC), A Coruña, 15001 Galicia, Spain; 11 Otolaryngology Unit, Santi Paolo e Carlo Hospital, Department of Health Sciences, Università degli Studi di Milano, 20121 Milan, Italy; 12 Department of Otolaryngology-Head and Neck Surgery, Foch Hospital, School of Medicine, UFR Simone Veil, Université Versailles Saint-Quentin-en-Yvelines (Paris Saclay University), 75000 Paris, France; prhans.foch@gmail.com; 13 Service of Otolaryngology, ENT Deparment, Virgen de la Macarena University Hospital,41000 Seville, Spain; 14 Department of Head and Neck Surgery, David Geffen School of Medicine, University of California Los Angeles, Los Angeles, CA 94305, USA; otoproof@gmail.com; 15 Department of Otolaryngology-Head and Neck Surgery, Stanford University School of Medicine, Stanford, CA 94305, USA; holsinger@stanford.edu; 16 Departamento de Otorrinolaringologia e Cirurgia de Cabeça e Pescoço, Universidade Federal de São Paulo (UNIFESP), 06000 São Paulo, Brazil; fpceccon@uol.com.br (F.P.C.); giannicolaiannella@hotmail.it (L.H.); 17 Department of Medical and Surgical Sciences and Advanced Technologies “GF Ingrassia” ENT Section, University of Catania, 95123 Catania, Italy; igolama@gmail.com (I.L.M.); s.cocuzza@unict.it (S.C.); fede.guli3@gmail.com (F.G.); 18 Division of Otolaryngology-Head and Neck Surgery, Center Hospitalier de l’Université de Montréal, Head and Neck Deparment, Montreal, QC 54550, Canada; 19 Department of Otolaryngology and Head and Neck Surgery, Foch Hospital, Paris Saclay University, 75000 Paris, France; 20 Department of Otolaryngology and Head and Neck Surgery, Division of Broncho-Esophagology, EpiCURA Hospital, UMONS Research Institute for Health Sciences and Technology, University of Mons (UMons), 7031 Mons, Belgium; 21 Department of Otolaryngology and Head and Neck Surgery, Elsan Polyclinic of Poitiers, 86000 Poitiers, France

**Keywords:** transoral, robotic, otolaryngology, head and neck surgery, survey, awareness

## Abstract

Background: To investigate perception, adoption, and awareness on the part of otolaryngology and head and neck surgeons (OTO-HNS) of transoral robotic surgery (TORS). Methods: Several items assessed: awareness/perception; access to TORS; training; indications and advantages/hurdles to TORS practice. A subanalysis was performed to assess differences according to the identified otolaryngological subspecialties. Results: A total of 359 people completed the survey. Among subspecialties, while for otolaryngologists 30/359 (8.4%) and H&N surgeons 100/359 (27.9%) TORS plays an effective role in hospital stay, laryngologists frequently disagreed (54.3%). There was a lower incidence among rhinologists and otologists (1.9%). Pediatric surgeons (0.8%) reported a positive response regarding the adoption of robotic surgery, and head and neck specialists expressed an even greater response (14.2%). Low adherence was related to perceived cost-prohibitive TORS, by 50% of H&N surgeons. Conclusions: Perception, adoption, and knowledge about TORS play a key role in the application of the robotic system, significantly varying across subspecialties.

## 1. Introduction

The use of transoral robotic surgery (TORS) has steadily grown over the past decade in the field of otolaryngology and head and neck surgery (ear, nose, and throat or ENT), introducing different innovations in surgical treatment approaches [1,2,3]. The contribution and value of TORS in head and neck oncologic surgery is now well established through robust clinical evidence, allowing a minimally invasive endoscopic approach with superior visualization and access compared to conventional transoral techniques for selected tumors such as oropharyngeal, parapharyngeal space, and laryngeal supraglottic carcinomas [4,5]. This has enabled improved resection and reconstruction in difficult-to-reach anatomical sites. Additionally, TORS has allowed for improved aesthetic and cosmetic outcomes through alternative surgical access routes that avoid external incisions; this has been most impactful and widely adopted in thyroid surgery procedures [6,7,8]. The dual benefits of improved clinical outcomes and functional results have driven rapid TORS adoption for specific ENT pathologies where its advantages over traditional external approaches are clearest. Ongoing technological refinements and expanded applications to other otolaryngologic subspecialties continue to spread TORS utilization. However, significant barriers to widespread dissemination remain, highlighting the importance of assessing current perceptions, familiarity, and access among the global ENT community to strategically guide optimal TORS integration. In 2015, the American Academy of Otolaryngology-Head and Neck Surgery Robotic Surgery Task Force proposed several recommendations to implement wider dissemination of and training for the TORS technique [9]. However, it is relevant to note that adoption of surgical robots remains less common in otolaryngology compared to other specialties, such as urology, general surgery, and thoracic surgery, where robotic platforms are used extensively [10,11,12]. Within ENT, TORS applications remain concentrated in head and neck oncologic procedures. The more limited use of robotics in ENT is related to structural limitations restricting broader access to the robotic platforms themselves. The immense costs of initial acquisition and ongoing annual maintenance, often over $100,000 per year, necessitates optimization of the timing and frequency of robotic use through coordination of schedules and sharing of capital investment among different surgical specialties at an institution [13,14]. This financial barrier can delay or prevent adoption at smaller centers lacking the procedural volume in each specialty to justify the expenditures. Strategic collaboration between specialties to maximize utilization of expensive robotic systems, collaborative cross-training programs between academic and community surgeons, and enhanced educational initiatives to increase TORS familiarity could help address these barriers and spread adoption more rapidly within otolaryngology. However, the unique cost constraints of surgical robotics remain a key challenge. Additionally, there are perceived barriers beyond just costs that have limited the application of TORS more extensively in ENT surgery. These include time constraints related to initial robotic system setup and prolonging operative docking time, lack of adequately trained operating room personnel familiar with the technology, and overall low volumes of TORS procedures performed at many centers that prevent surgeons from gaining proficiency [15,16,17]. Awareness of and receptiveness to adopting TORS on the part of ENT surgeons is also often negatively influenced by incomplete knowledge of the most appropriate evidence-based indications for robotic surgery or lack of familiarity with the actual benefits related to the TORS approach. It is also hampered by the lack of adequate TORS training programs and pathways to gain hands-on experience [18,19,20]. Although issues related to cost-benefit analysis, training barriers, and general skepticism toward new surgical technologies have been addressed previously in the literature, there is currently no large-scale survey specifically evaluating the holistic perception, awareness, utilization patterns, and adoption of TORS among ENT surgeons across subspecialties. Nor do prior studies capture international perspectives on TORS. Understanding the nuanced specialty- and region-specific factors influencing TORS adoption can inform targeted interventions to address knowledge gaps, provide training, and align economic incentives to drive uptake where clinical benefits are clearest based on procedure volume; support infrastructure is also key for successful adoption. This international survey aimed to thoroughly investigate the multitude of factors that influence the perception, utilization, and overall adoption of TORS across the broad field of ENT surgical practice, encompassing all subspecialty groups. By gathering perspectives from a diverse global sample of otolaryngologists at various career stages, practice settings, and geographic regions, this study sought to elucidate the key drivers and barriers shaping acceptance of TORS technology. Factors including cost, training, procedure volume, infrastructure, surgeon experience, specialty-specific procedural needs, and regional practice patterns were explored through focused survey questions. The goal was to produce a comprehensive snapshot of the current TORS landscape worldwide across the ENT community, in order to inform future efforts to optimize dissemination and training. Targeted interventions to promote adoption where evidence and resource availability warrant can then be tailored to the needs of specific subgroups, leading to more thoughtful TORS integration. These data are key to guide the evolution of surgical robotics in ENT, urology, and other specialties undergoing this technology transition.

## 2. Materials and Methods

### 2.1. Survey Design

The Young Otolaryngologist Robotics Group of the International Federation of Otolaryngology Societies (YO-IFOS) created the survey including in the protocol otolaryngology surgeons and robotic experts from all continents. Eighteen items were included in the final version of the survey: demographic information (n = 5); experience and practice of TORS (n = 3); training (n = 2); access (n = 1); perception of TORS (n = 1); barriers/disadvantages/benefits (n = 2); indications (n = 1); setting (n = 2); and improvements (n = 1). The item regarding TORS indications provided a 5-point scale ranging from “No indication” (0) to “Perfect indication” (5). Benign neck tumors, thyroid surgery, sleep apnea surgery, and oropharyngeal, laryngeal, hypopharyngeal, and nasopharyngeal malignancies were included among the listed conditions. Because the survey did not report sensitive data on the patients or on the experiments performed, no Institutional Review Board committee was provided.

### 2.2. Survey Spread

The survey was based on an electronic questionnaire designed with the SurveyMonkey^®^ software (SurveyMonkey Inc., version 4.1.1, San Mateo, CA, USA) and allowed only one response per study participant. The survey was emailed to a list of 1383 comprising YO-IFOS/IFOS members from Europe, North America, South America, East and West Asia, Oceania, and Africa.

### 2.3. Collection and Data Analysis

The data were collected anonymously for each participant. Incomplete responses were excluded from the final analysis. As a first step, responses were analyzed for the entire cohort of participants. Subsequently, a subanalysis was performed focusing on the 6 subspecialty groups: Laryngology, Head & Neck, General and Pediatric Otolaryngology, Rhinology, Otology, and Residency. We performed the statistical analyses with the Statistical Package for the Social Sciences for Windows (SPSS version 22.0; IBM Corp, Armonk, NY, USA). Differences in response between the groups were assessed using the Kruskal–Wallis test or the χ2 test, depending on the data type.

## 3. Results

### 3.1. Study Participants

A total of 359 otolaryngologist respondents completed the survey. Among them, most reported working primarily in an academic university hospital setting (n = 208/359—58%), while a smaller proportion worked mainly in private practice centers (n = 50/359—14%) or split their time between academic and private settings (n = 101/359—28%). In terms of subspecialty breakdown, the most common groups represented were general ENT physicians (n = 47/359—13.1%), head and neck surgical oncologists (n = 170/359—47.35%), laryngologists (n = 35/359—9.74%), and rhinologists/otologists (n = 56/359—15.59%). Additional responses came from pediatric otolaryngologists (n = 20/359—5.57%) and otolaryngology residents in training (31/359—8.63%). The wide range of subspecialties provides useful perspectives across the breadth of ENT surgery. The high proportion of academic practitioners likely reflects greater access to advanced technologies like TORS at university hospitals currently. However, the survey also captured insights from private practice respondents to understand a diversity of settings. The subgroup analysis by practice type and subspecialty reveals how opinions on TORS vary across the ENT field based on surgical focus, resources, and exposure (Table 1).

### 3.2. Perception, Barriers, and Benefits

Among the enrolled surgeons, there is an optimistic perception of the TORS procedure, with 57.7% (N = 207) believing that TORS procedures result in a reduction in the length of hospital stay, 59.6% (N = 214) believing that it improves the postoperative patient’s quality of life and 57.7% (N = 207) believing that it allows avoiding tracheotomy in selected cases. However, on subspecialty analysis, ENT general (30/47—63.8%) and H&N surgeons (100/170—58.8%) believe that TORS has an effective role in hospital stay, while laryngologists expressed an opposite opinion (19/35—54.3%). Contrasting data were also expressed on the actual cosmetic benefit via alternative surgical access routes allowed by TORS, with 188/359 (52.4%) of the participants not perceiving an improvement, predominantly among H&N surgeons (25.6%) and rhinologists and otologists (8.4%). The perceived feasibility in surgical practice of the robotic system was found to vary according to the surgical site such as the oropharynx, hypopharynx, and larynx, and on the stage of pathology at diagnosis. For early-stage oropharyngeal carcinomas, TORS was found to be applicable by 88.2% of the respondents, especially for H&N surgeons (n= 154/170—89.9%) and laryngologists (n = 32/35; 91.5%). However, the indication dropped dramatically for cT3 oropharyngeal carcinoma for both H&N subspecialties (67/170; 33.6%) and laryngologists (14/34; 41.1%). In contrast, pediatric (15/19; 89% vs. 11/19; 57.9%) and general (40/47; 85.1% vs. 29/47, 51.7%) surgeons maintained a large percentage in the indication for therapy, although decreased. This reduction in indication also occurred for supraglottic cT1-2 vs. cT3 tumors among specialists such as laryngologists (31/35 88.6% vs. 9/34; 26.5%), rhinologists, and otologists (48/55; 87.3% vs. 17/53; 32.1%).

### 3.3. Awareness and Opinion of TORS

Awareness of the different benefits provided by TORS was present among most of the participants (n = 217/359, 60.4%), believing that the method represents the future of minimally invasive surgery in otolaryngology–head and neck surgery (n = 178/359; 49.6%). This awareness is especially rooted among H&N surgeons (n = 88/170—51.8%), rhinologists and otologists (n = 27/56—48.21%), and laryngologists (n = 21/35—11.8%). However, the first approach to robotic surgery appeared challenging, reporting an overall low level of optimism (n = 83/359—23.1%), especially among rhinologists and otologists (n = 7/56; 12.5%) and pediatricians (n= 3/20—15%). In contrast to other subspecialties, general ENT surgeons approached TORS more optimistically in their initial experience (n= 51/170—30%). However, this openness to adopting robotic approaches did not always translate into a willingness to actually recommend its widespread adoption in surgical practice. There was an overall reluctance among participants to advocate for broader TORS adoption overall (n = 257/359; 71.6%), an attitude especially prevalent among rhinologists and otologists (n= 45/56; 80.4%), pediatric ENTs (n = 15/20—75%), and general ENT surgeons (n = 34/47—72.3%). In contrast, a greater proportion (n = 14/35—40%) of laryngologists expressed a more favorable opinion about actively spreading the TORS method among colleagues. This reluctance may stem from the fact that the vast majority of participants (n = 337/359—93.9%) believe there are more disadvantages and barriers than advantages to TORS currently. This perspective resulted in an unwillingness on the part of many respondents to refer appropriate robotic surgery candidates to other centers with TORS programs. Most participants (206/359—57.4%) still preferred to perform procedures through traditional endoscopic or open surgical approaches with which they are more comfortable. This preference for conventional surgery was expressed by 40% of pediatric ENTs, 57.1% of laryngologists, and 50% of rhinologists and otologists. Thus, while awareness of TORS benefits is high, skepticism regarding its advantages vs. drawbacks prevails across most subspecialties. Targeted education on appropriate indications, hands-on training, and warm referrals may help increase adoption. But overcoming ingrained preferences for the status quo remains challenging.

### 3.4. Adoption, Accessibility, and Cost Perception

Greater adequate access among TORS participants and adoption in clinical practice was reported in (116/359—32.3%) responses, including (78/170—45.9%) belonging to H&N subspecialty and laryngologists (13/35—37.1%). Also, among participants who did not have access to TORS (141/359—39.3%), most reported interest in adopting it, including in particular H&N surgeons (54/170—31.8%) and rhinologists and otologists (28/56—50%). In contrast, complete lack of interest in TORS was poorly shown despite not accessing the method (34/359—9.5%), most of all among general surgeons (7/47—14.9%), H&N surgeons (8/170—4.7%), and rhinologists and otologists (9/56—16%). Low adherence was rarely correlated with a perception of cost-prohibitive TORS (n = 6/359—1.7%). Yet the availability of the robotic system is perceived as a barrier to its use in 262/359 (73%), especially among laryngologists n = 29 (82.9%), rhinologists and otologists n = 43 (76.8%) and pediatricians n = 18 (90.0%). Economic expenditure significantly impacts the adoption of the robotic system, reaching up to 72.3% of ENT generals, 73.2% of rhinologists and otologists, and 80% of pediatricians. In contrast, the timing related to the adoption of the robot was not considered a limitation to its use, achieving 279 (77.7%) favorable responses. However, some important nuances emerged when looking at the subanalysis of responses by subspecialty. Despite the generally enthusiastic view of TORS in the full sample, laryngologists and pediatric otolaryngologists still expressed some disfavor, with 28.6% and 25% of respondents in those groups, respectively, reporting a negative perception of the value of TORS in their field. Similarly, overall lack of interest in adopting TORS did not play a major role as a barrier, but it was reported in a noteworthy minority of cases—14.9% among general ENT surgeons and 15% in pediatric ENTs. No laryngologists expressed outright lack of interest, but their subspecialty had the most negative perceptions overall. Although the TORS robotic system is often considered overly complex and prolonged in initial setup time (often 30–60 min to position the console and articulated arms) and added operative docking time (up to 15 min for robot positioning and trocar placement) compared to conventional endoscopic procedures, and its bulk can limit exposure and maneuverability in the narrow surgical field, participants in our survey reported an overall optimistic approach to both of these issues. 86.6% felt that lengthy setup times were a manageable barrier, and 78.8% felt that limitations in exposure did not preclude sufficient application of TORS in appropriate cases. However, in the subanalysis among specialties, some differences emerged. Difficult surgical exposure was reported as a key limitation predominantly in pediatric ENT surgeons (30%) and head and neck oncologic surgeons (25.3%), likely due to the smaller anatomy in children and space-limiting tumors, while issues with docking and setup time were noted more often among rhinologists and otologists (17.9%) as well as pediatric ENTs (20%), perhaps due to smaller sinonasal spaces and airway openings in their typical procedures. This highlights that while broad enthusiasm exists for adopting TORS in appropriate cases, certain subspecialties have unique procedural concerns and restrictions that may limit universal applicability. A nuanced approach tailored to each group will be essential as TORS becomes more widespread.

## 4. Discussion

This survey is the first international cross-sectional study evaluating the perception of subspecialty otolaryngologists toward TORS, and it aims to fill an important gap in understanding this emerging robotic technique. The primary objective was to comprehensively assess the current perception, adoption rates, and overall awareness of TORS among the different subspecialties of otolaryngology and head and neck surgery. Although TORS procedures were used more frequently in certain areas of the otolaryngology specialty, such as head and neck oncology, there were still substantial barriers to wider application across all subspecialties, related not only to economic factors like high costs of purchasing and maintaining robotic systems, but also to differences in surgeons’ trust, confidence, comfort level, familiarity, and willingness to adopt new TORS procedures into their practices [21]. Gaining a detailed understanding of the multifaceted barriers and specialty-specific nuances shaping TORS perceptions is critical to inform targeted educational and advocacy efforts to appropriately disseminate this technology where evidence supports patient benefit. This international survey provides initial data to guide wider adoption while identifying concerns that must be thoughtfully addressed before TORS is accepted more universally. Our survey found an overall optimistic perception of the TORS procedure among the participants across multiple subspecialties. Potential benefits like shortened hospital stays (reported by 57.7% of respondents), improved patient quality of life in the postoperative period (59.6%), and avoiding tracheotomies in selected cases (57.7%) were frequently cited as advantages by ENT surgeons and head and neck oncologists. This generally enthusiastic view among the survey respondents seems somewhat incongruous with the widespread opinion in the literature that the use of the robotic system significantly increases upfront costs and long-term economic healthcare expenditures. The high fixed prices of purchasing and installing the large, sophisticated robotic systems, which can cost over $2 million, as well as the recurring costs associated with proprietary disposable instruments, annual servicing/maintenance contracts, additional operating room time given longer procedure durations, and specialized staff training often influence the choice of surgical approach. These substantial costs frequently lead hospital administrators and surgeons to favor more affordable conventional endoscopic or open techniques over the more expensive TORS approach based on cost-effectiveness analyses. However, in contrast to these expectations based on the high absolute costs documented in health economics studies, our survey reported that low adherence to adopting TORS was rarely related to its being perceived as prohibitive in cost by the responding surgeons themselves. This reveals a disconnect between the objective TORS costs cited in the literature and surgeons’ subjective perceptions of affordability barriers, suggesting that the benefits of the approach are viewed as outweighing the costs for appropriate patients. Delving deeper into this perception gap, factors like underestimating total systems costs, lack of personal responsibility for budgetary constraints as employees of large hospitals, and emphasis on clinical benefits over economic factors may skew surgeons’ perspectives on TORS costs compared to administrators tasked with budget management. Additionally, the common desire to adopt new technologies and provide the best care with cutting-edge tools may influence surgeons’ willingness to downplay real-world costs. Further research is needed to better align subjective surgeon perceptions of TORS costs and benefits with rigorous real-world hospital cost-effectiveness data. This will help generate evidence-based protocols for efficient TORS adoption that balance patient outcomes and judicious resource utilization. Only a small percentage (1.7%) of total respondents across all subspecialties cited cost as a major barrier to adoption, including just 50% of head and neck cancer surgeons. This finding reveals a disconnect between actual TORS costs and surgeons’ perceptions of the affordability barriers. It suggests an optimism that the approach’s benefits outweigh the costs in appropriate patients. However, upon subspecialty analysis, this perception showed mixed results. While general ENT specialists (8.4%) and head and neck surgeons (27.9%) saw TORS as potentially decreasing hospital stays and improving outcomes sufficiently to warrant the costs, laryngologists were strongly against TORS for cost reasons in up to 54.3% of cases. Within the same field of otolaryngology, subspecialty nuances exist in the perceived cost-benefit tradeoffs of TORS adoption. This variability highlights the need for a nuanced understanding of specialty-specific perspectives on the costs and benefits of TORS to gain wider adoption while addressing lingering economic concerns. Blanket approaches to TORS advocacy must give way to tailored messaging and training that resonates with each subfield’s clinical and financial realities. Head and neck surgeons already adept at complex oncological procedures may be more enthusiastic about TORS potential despite the costs, while smaller-scope specialties like laryngology may remain unconvinced. Continued outcome data collection and cost-effectiveness analyses could help provide the evidence to overcome objections on a case-by-case basis. However, adoption will only grow through open dialog, education, and willingness to understand alternative viewpoints across diverse otolaryngology subspecialties. Although fascinating and innovative, the first impact of the TORS procedure may not be positive for all surgeons, leading some specialists to have no interest in this technology [22]. Probably, this may be due to the still inadequate dissemination and lack of accessibility of robotic programs, which to date remain present only in well-funded academic centers with specialized teams of highly experienced head and neck surgeons and substantial hospital financial support for the acquisition and maintenance of expensive robotic systems [13,14,19]. This concept is in line with our survey data, as an almost global opinion was reported among participants (93.9%) that TORS, while promising, was a method with many disadvantages and barriers to widespread adoption currently, including issues reaching 97.9% of general-ENT respondents, 95% of pediatric ENTs, and even 100% of laryngologists. Mandapathil et al. reported a correlation between poor acceptance of TORS and lack of collaboration between early-adopting academic centers and community hospitals or practices [21]. Without adequate hands-on training opportunities and sharing of acquired surgical experience, TORS is inconsistent with acceptance and adoption for new surgical indications. However, robotic surgery has found wide development of applications in specific areas, especially in the field of head and neck oncologic surgery, where through less invasive approaches enabling improved access and visualization, treatment of oropharyngeal or supraglottic stage I–II cancers has shown promising outcomes [23,24,25]. Yet for broader dissemination beyond specialized academic centers, advanced techniques like TORS that are pioneered at well-funded institutions must gradually disseminate to community practitioners through collaborative training programs and robotic systems sharing arrangements to overcome the sizable barriers to universal adoption. Hands-on training and mentoring from experienced TORS surgeons will be key to increasing uptake. This pattern found in the literature was confirmed by our subanalysis, where greater adequate access to and clinical adoption of TORS was found among survey respondents belonging to the head and neck oncologic surgery subspecialty (45.9%) and laryngologists (37.1%)—groups that are concentrated in academic medical centers. The same confidence in the method and interest in future adoption was also shown in respondents without current access to TORS systems, specifically head and neck surgeons in private practice (38.3%) and community-based rhinologists and otologists (19.9%). This demonstrates an openness to adopting TORS more widely if barriers like costs, training, and accessibility can be addressed through cross-collaboration between specialty groups and practice settings. Maximizing this willingness through creative solutions could drive faster TORS dissemination. In contrast, 9.5% of cases showed no interest in TORS while not accessing the method, especially among general surgeons (20.6%). Chen et al. demonstrated a significant increase in the robotic system, up to 67%, in US academic centers with adequate training programs, correlating the method with better postoperative outcomes [19]. Our survey confirmed that the willingness to refer patients with robotic indication to other provided centers did not occur in 57.4% of the participants, who preferred to perform procedures through traditional endoscopic or open surgical approaches. In particular, this preference was expressed by pediatricians (40%), laryngologists (57.1%), rhinologists, and otologists (50%). Of course, both the habits and the experience (training) of the surgeon seem to play a major role in the adoption of a new procedure [16,17,18]. A relevant aspect that could be a barrier to TORS is that the robotic system is considered to be artificial and prolonged in both setup and docking time. Moreover, surgical field exposure is often considered a limitation to the applicability of the method, which could be more difficult in some patients than conversion to a traditional approach. In contrast, our survey showed that among the participants both the factors of setting and difficult exposure are not perceived as problematic to feasibility (86.6% and 78.8%, respectively). However, this conception is variable in the subanalysis, where we noted that difficult exposure was a limitation predominantly for pediatricians (30%) and H&N (25.3%), while difficult docking/setting was a limitation predominantly for rhinologists and otologists (17.9%). The present study has some important limitations that must be acknowledged. First, the relatively small number of total participants must be considered the primary limitation. Although we strived to conduct a large global survey-based study on TORS perceptions spanning several world regions, the total sample size remains small at just over 200 respondents. The low number may be partly attributed to the lack of access to TORS robotic systems in some areas, including many countries in Eastern Europe, Asia, Africa, and South America, limiting first-hand experience in these regions. Secondly, the very poor representation of surgeons from Africa, the Middle East, and Oceania may be motivated not only by fewer active members in the international scientific society involved in the present study in these areas but also by generally lower absolute numbers of practicing otolaryngologists and extremely limited access to advanced surgical robotics technology in these under-resourced regions. This significantly skews the worldwide results toward Western perspectives. Additional limitations include potential response bias among those most interested in TORS, lack of detail on the specific procedures surveyed, and inability to assess evolution over time as TORS becomes more widespread. In the future, targeted outreach to expand participation globally, follow-up surveys after broader TORS dissemination, and qualitative research on motivations could help overcome these limitations and provide a richer understanding of the nuances around TORS perceptions and adoption worldwide. However, this initial study provides a useful baseline snapshot of the current landscape.

## 5. Conclusions

The present study exposed TORS perception, adoption, and awareness among different ENT subspecialties. Variables such as the anatomical area involved, tumor staging, and the surgeon’s experience with TORS influence the adoption, perception, and use of TORS in otolaryngology–head and neck surgery. Our findings yield information necessary to increase awareness and adoption of the procedure among different ENT subspecialties, even in centers without access to TORS.

## Figures and Tables

**Table 1:** Main demographic features of the participants.

Outcomes	All (359)
Gender (F/M)	96/263
Year of experience (years)	15.6 ± 14.4
World regions	
Europe	120 (33%)
North America	35 (10%)
Asia	96 (27%)
South America	84 (23%)
Africa	16 (4%)
Oceania	8 (2%)
Places of practice	
Academic/University	209 (58%)
Private	50 (14%)
Academic and private	100 (28%)
ENT subspecialty	
General	47/359 (13.1%)
Head and Neck	170/359 (47.35%)
Laryngologist	35/359 (9.74%)
Otologists and Rhinologists	56/359 (15.59%)
Pediatricians	20/359 (5.57%)
Residents	31/359 (8.63%)

Among the most represented backgrounds were Europeans (120/359—33%), Asians (96/359—27%), and South Americans (84/359—23%).

## Data Availability

No new data were created on patient features due to study design.

## References

[1] McLeod I.K., Mair E.A., Melder P.C. (2005). Potential Applications of the da Vinci Minimally Invasive Surgical Robotic System in Otolaryngology. Ear Nose Throat J..

[2] Sobel R.H., Blanco R., Ha P.K., Califano J.A., Kumar R., Richmon J.D. (2016). Implementation of a comprehensive competency-based transoral robotic surgery training curriculum with ex vivo dissection models. Head Neck.

[3] Bhayani M.K., Holsinger F.C., Lai S.Y. (2010). A shifting paradigm for patients with head and neck cancer: Transoral robotic surgery (TORS). Oncology.

[4] Meccariello G., Maniaci A., Bianchi G., Cammaroto G., Iannella G., Catalano A., Sgarzani R., De Vito A., Capaccio P., Pelucchi S. (2021). Neck dissection and trans oral robotic surgery for oropharyngeal squamous cell carcinoma. Auris Nasus Larynx.

[5] Weinstein G.S., O’Malley B.W., Cohen M.A., Quon H. (2010). Transoral Robotic Surgery for Advanced Oropharyngeal Carcinoma. Arch. Otolaryngol. Neck Surg..

[6] Othman S., McKinnon B.J. (2018). Financial outcomes of transoral robotic surgery: A narrative review. Am. J. Otolaryngol..

[7] Iseli T.A., Kulbersh B.D., Iseli C.E., Carroll W.R., Rosenthal E.L., Magnuson J.S. (2009). Functional outcomes after transoral robotic surgery for head and neck cancer. Otolaryngol. Neck Surg..

[8] Tae K., Song C.M., Ji Y.B., Sung E.S., Jeong J.H., Kim D.S. (2016). Oncologic outcomes of robotic thyroidectomy: 5-Year experience with propensity score matching. Surg. Endosc..

[9] Gross N.D., Holsinger F.C., Magnuson J.S., Duvvuri U., Genden E.M., Ghanem T.A., Yaremchuk K.L., Goldenberg D., Miller M.C., Moore E.J. (2016). Robotics in otolaryngology and head and neck surgery: Recommendations for training and credentialing: A report of the 2015 AHNS education committee, AAO-HNS robotic task force and AAO-HNS sleep disorders committee. Head Neck.

[10] Bhandari M., Zeffiro T., Reddiboina M. (2020). Artificial intelligence and robotic surgery: Current perspective and future directions. Curr. Opin. Urol..

[11] Chang T.C., Seufert C., Eminaga O., Shkolyar E., Hu J.C., Liao J.C. (2021). Current Trends in Artificial Intelligence Application for Endourology and Robotic Surgery. Urol. Clin. N. Am..

[12] Morrell AL G., Morrell-Junior A.C., Morrell A.G., Mendes J., Freitas M., Tustumi F., Morrell A. (2021). The history of robotic surgery and its evolution: When illusion becomes reality. Rev. Colégio Bras. Cir..

[13] Liberman D., Trinh Q.D., Jeldres C., Zorn K.C. (2012). Is robotic surgery cost-effective: Yes. Curr. Opin. Urol..

[14] Byrd J.K., Paquin R. (2020). Cost Considerations for Robotic Surgery. Otolaryngol. Clin. N. Am..

[15] Bertolo R., Garisto J., Gettman M., Kaouk J. (2018). Novel System for Robotic Single-port Surgery: Feasibility and State of the Art in Urology. Eur. Urol. Focus.

[16] Ghazanfar S., Qureshi S., Zubair M., Fateh U., Ahmed S., Quraishy M.S. (2019). Feasibility of robotic surgery in a developing country, a public sector perspective. JPMA.

[17] Dhanani N.H., Olavarria O.A., Millas S., Askenasy E.P., Ko T.C., Liang M.K., Holihan J.L. (2020). Is robotic surgery feasible at a safety net hospital?. Surg. Endosc..

[18] Castaldi M., Palmer M., Con J., Abouezzi Z., Latifi R., Bergamaschi R. (2021). Robotic-Assisted Surgery Training (RAST) Program: An Educational Research Protocol. Surg. Technol. Int..

[19] Chen R., Force S.R.T., Armijo P.R., Krause C., Siu K.-C., Oleynikov D. (2019). A comprehensive review of robotic surgery curriculum and training for residents, fellows, and postgraduate surgical education. Surg. Endosc..

[20] Moit H., Dwyer A., De Sutter M., Heinzel S., Crawford D. (2019). A Standardized Robotic Training Curriculum in a General Surgery Program. JSLS J. Soc. Laparosc. Robot. Surg..

[21] Mandapathil M., Meyer J.E. (2021). Acceptance and adoption of transoral robotic surgery in Germany. Eur. Arch. Oto-Rhino-Laryngol..

[22] O’Malley B.W., Weinstein G.S. (2007). Robotic skull base surgery: Preclinical investigations to human clinical application. Arch. Otolaryngol. Head Neck Surg..

[23] Chan J.Y., Tsang R.K., Eisele D.W., Richmon J.D. (2015). Transoral robotic surgery of the parapharyngeal space: A case series and systematic review. Head Neck.

[24] Byeon H., Holsinger F., Kim D., Kim J., Park J., Koh Y., Choi E. (2014). Feasibility of robot-assisted neck dissection followed by transoral robotic surgery. Br. J. Oral Maxillofac. Surg..

[25] Krishnan S., Connell J., Ofo E. (2016). Transoral robotic surgery base of tongue mucosectomy for head and neck cancer of unknown primary. ANZ J. Surg..

